# Combining initial chest CT with clinical variables in differentiating coronavirus disease 2019 (COVID-19) pneumonia from influenza pneumonia

**DOI:** 10.1038/s41598-021-85779-1

**Published:** 2021-03-19

**Authors:** Shuang Zhao, Zixing Huang, Hanjiang Zeng, Zhixia Chen, Fengming Luo, Chongwei Zhang, Bin Song

**Affiliations:** 1 Department of Radiology, West China Hospital, Sichuan University, Chengdu, Sichuan China; 2 Department of Respiratory and Critical Care Medicine, West China Hospital, Sichuan University, Chengdu, Sichuan China; 3 Department of Laboratory Medicine, West China Hospital of Sichuan University, Chengdu, Sichuan China

**Keywords:** Respiratory tract diseases, Diagnostic markers, Computed tomography, Infectious diseases, Influenza virus, Viral infection

## Abstract

Coronavirus disease 2019 (COVID-19) has spread in more than 100 countries and regions around the world, raising grave global concerns. COVID-19 has a similar pattern of infection, clinical symptoms, and chest imaging findings to influenza pneumonia. In this retrospective study, we analysed clinical and chest CT data of 24 patients with COVID-19 and 79 patients with influenza pneumonia. Univariate analysis demonstrated that the temperature, systolic pressure, cough and sputum production could distinguish COVID-19 from influenza pneumonia. The diagnostic sensitivity and specificity for the clinical features are 0.783 and 0.747, and the AUC value is 0.819. Univariate analysis demonstrates that nine CT features, central–peripheral distribution, superior–inferior distribution, anterior–posterior distribution, patches of GGO, GGO nodule, vascular enlargement in GGO, air bronchogram, bronchiectasis within focus, interlobular septal thickening, could distinguish COVID-19 from influenza pneumonia. The diagnostic sensitivity and specificity for the CT features are 0.750 and 0.962, and the AUC value is 0.927. Finally, a multivariate logistic regression model combined the variables from the clinical variables and CT features models was made. The combined model contained six features: systolic blood pressure, sputum production, vascular enlargement in the GGO, GGO nodule, central–peripheral distribution and bronchiectasis within focus. The diagnostic sensitivity and specificity for the combined features are 0.87 and 0.96, and the AUC value is 0.961. In conclusion, some CT features or clinical variables can differentiate COVID-19 from influenza pneumonia. Moreover, CT features combined with clinical variables had higher diagnostic performance.

## Introduction

A new coronavirus, severe acute respiratory syndrome coronavirus 2 (SARS-CoV-2), emerged in Wuhan, China, at the end of 2019. The World Health Organization (WHO) named the disease caused by this pathogen coronavirus disease 2019 (COVID-19). On March 11, 2020, the WHO declared COVID-19 a pandemic, and SARS-CoV-2 infection has since reached pandemic status worldwide. According to the latest statistics from the WHO, it brings the global cumulative numbers to 108.2 million confirmed cases of COVID-19, and over 2.3 million death cases since the start of the pandemic^1^.

In winter, influenza is more common than COVID-19. Influenza is a mild-to-severe, contagious respiratory illness caused by the influenza viruses. Serious outcomes include hospitalization or death, and the elderly, the young, and people with poor health conditions are at a high risk of these complications. The most common form of influenza is seasonal influenza. The two main types of seasonal influenza virus, A and B, are routinely spread among humans and are responsible for seasonal influenza epidemics each year. Influenza accounts for thousands of deaths and hospital admissions annually in the USA, with an even greater impact in developing countries^2–5^. COVID-19 and seasonal influenza pneumonia are similar in terms of transmission and symptoms. In both cases, the primary modes of transmission are droplets and contact transmission. Fever, chills, headache, myalgia, malaise, and anorexia accompanied by respiratory symptoms, including nonproductive cough and sore throat, are the main symptoms of both^6–8^. Reverse transcription polymerase chain reaction (RT-PCR) is a highly recommended diagnostic technique. However, some studies have indicated that PCR results are susceptible to false negatives from several factors^9–11^.

Some studies have shown that GGO, consolidation with air bronchogram, interlobular septal thickening, centrilobular nodules, and reticular opacities were the main CT manifestations of influenza pneumonia^12–14^. Many studies have revealed the CT features of COVID-19, such as GGO, vascular enlargement, interlobular septal thickening^15,16^. COVID-19 is also a viral pneumonia, and its CT features overlap with those of other viral pneumonias^12^. Recently, studies have compared the differences in chest CT findings between COVID-19 and influenza pneumonia^17,18^. Their study showed that some CT features of COVID-19 and influenza pneumonia were different. However, their study has some shortcomings, such as less clinical variables, no further analysis of which features have differential diagnostic performance, and no comprehensive analysis of CT features combined with clinical variables.

This study aimed to investigate the differential diagnosis between influenza pneumonia and COVID-19 based on CT features combined with clinical variables.

## Materials and methods

### Patients

This retrospective study was approved and the requirement for informed consent was waived by the Biomedical Research Ethics Committee of West China Hospital of Sichuan University. The study follows the principles of the Declaration of Helsinki with voluntary participation. The data are analyzed and handled in an anonymous format. We adhere to relevant guidelines and regulations in all experiments.

COVID-19 group: we reviewed the records of consecutive patients with COVID-19 confirmed by RT-PCR assay at our hospital from January 21st, 2020 to February 9th, 2020. Inclusion criteria were availability of complete data from CT examinations; complete medical record; and no coinfection; and absence of other lung diseases, tumor, hypertension, diabetes.

Seasonal influenza pneumonia group: we reviewed the records of consecutive patients with influenza virus pneumonia (types A and B) confirmed by nucleic acid testing at our hospital from January 21st, 2020 to February 9th, 2020. Inclusion criteria were the same as those for patients with COVID-19 pneumonia.

The clinical variables included sex, age, exposure history (Wuhan travel history or close contact with confirmed patients with COVID-19), smokers, alcoholics, days from illness onset, temperature, respiratory rate, systolic pressure, diastolic pressure, oxygen saturation, and symptoms (fever, rigor, chills, cough, sputum production, chest, tightness, dyspnea, sore throat, fatigue, headache, myalgia, abdominal pain, and diarrhea).

### CT image acquisition

Chest CT was performed on a 128-slice CT scanner (Revolution CT Scanner; GE Medical Systems, Milwaukee, Wisconsin, USA) dedicated only to patients with COVID-19. The CT protocol was: tube voltage, 120 kV (automatic adjustment); tube current, 200–500 mAs; rotation time, 0.5 s; section thickness, 0.625 mm; collimation, 0.625 mm; pitch, 1; matrix, 512 × 512; and inspiration breath hold.

Patients with seasonal influenza pneumonia were imaged by a 128-slice CT scanner (Somatom Definition AS + CT scanner; Siemens Healthcare, Forchheim, Germany). The CT protocol was: tube voltage, 120 kV; tube current, 110 mAs (automatic adjustment); rotation time, 0.5 s; section thickness, 0.75 mm; collimation, 0.6 mm; pitch, 1; matrix, 512 × 512; and inspiration breath hold.

All scans were obtained with the patient in the supine position during end-inspiration without intravenous contrast material. Reconstruction was performed with a bone algorithm with a thickness of 1 mm and an interval of 1 mm. The following windows were used: a mediastinal window with a window width of 350 HU and a window level of 40 HU and a lung window with a width of 1800 HU and a level of − 400 HU.

### CT image review

All CT images were reviewed by two fellowship-trained cardiothoracic radiologists with approximately 10 years of experience each. Each radiologist was blinded to laboratory assay results and demographic information including date of CT examination and patient name. Images were reviewed independently, and final decisions were reached by consensus. Disagreements were resolved by a third fellowship-trained cardiothoracic radiologist with 15 years of experience.

The distribution of lung abnormalities was recorded as: (a) left, right or bilateral lung; (b) predominantly subpleural (involving the peripheral 1/3 of the lung), hilar (involving around the hilum), and random (without predilection for subpleural or around hilum); (c) predominantly superior (superior to the bifurcation of trachea), inferior (inferior to the bifurcation of trachea), and random (without predilection for superior or inferior); (d) predominantly anterior (anterior to the horizontal line across the axillary midline), posterior (posterior to the horizontal line across the axillary midline), and random (without predilection for anterior or posterior); (e) presented as a solitary lesion. Numbers of involved lobes and segments of lungs were recorded also.

The CT findings included ground-glass opacity (GGO), consolidation, crazy-paving pattern, bronchiolectasis, interlobular septal thickening, and lymphadenopathy. Other abnormalities were noted; for example, cavitation, air bronchogram, reticulation, calcification, subpleural curvilinear line, halo sign, pleural effusion, pleural thickening, and pericardial effusion.

### Statistical analysis

All statistical analyses were performed with SPSS 20.0 (IBM, Armonk, NY, USA). Student’s t-tests or Mann–Whitney U were used to compare two groups according to the data distribution. Chi-square test or Fisher’s exact test was used for the two categorizable comparison. In addition, variables for which more than 30% of values were missing were not included in logistic regression analysis. Firstly, univariate logistic analysis was used for the selection of these variables, and then, those significantly variable in univariate analysis were used for further multivariate logistic analysis by using a stepwise method. For all variables, odds ratios (OR) and 95% confidence intervals (95% CI) were calculated, and, then, diagnostic sensitivity, specificity, and area under the curve (AUC) were determined. A two-tailed p value less than 0.05 was considered statistically significant.

### IRB statement

This retrospective study was approved by the Biomedical Research Ethics Committee of West China Hospital of Sichuan University. The requirement for informed consent was waived, given the retrospective nature of the study.

## Results

### The clinical variables

Twenty-four patients with COVID-19 (mean age, 41 years; 13 men) and 79 patients with influenza pneumonia (mean age, 41 years; 50 men) were enrolled in the study. They were all newly diagnosed patients. The onset time is about 1 week. There were significant differences in exposure history, temperature, respiratory rate and systolic blood pressure between the two groups, but no differences in other variables (Table 1). The temperature and respiratory rate of patients with influenza pneumonia were higher than patients with COVID-19, while the systolic blood pressure was lower than patients with COVID-19.

**Table 1:** The clinical variables in 79 patients with influenza pneumonia and 24 with COVID-19.

Variables	Influenza pneumonia (*n* = 79)	COVID-19 (*n* = 24)	*p*
Sex, m/f	50/29	13/11	0.48
Age mean (SD) (years)	40.9 (19.5)	46.17 (17.4)	0.12
Exposure history	0	24	0.000
Smokers, n (%)	12 (15.19)	2 (8.33)	0.49
Alcoholics, n (%)	13 (16.46)	2 (8.33)	0.50
Days from illness onset mean (SD)	7.04 (8.85)	5.91 (6.79)	0.84
Temperature, mean (SD) (°C)	38.14 (1.12)	37.61 (0.86)	0.04
Respiratory rate, mean (SD) (bpm)	20.70 (2.07)	19.92 (1.50)	0.02
Systolic pressure, mean (SD) (mmHg)	128.58 (15.81)	137.71 (14.26)	0.03
Diastolic pressure, mean (SD) (mmHg)	80.29 (14.38)	85.17 (14.76)	0.08
Oxygen saturation, n (%)	96.02 (3.39)	96.79 (1.38)	0.78
**Symptom, n (%)**			
Fever	61 (77.22)	17 (70.83)	0.666
Rigor	8 (10.13)	0	0.246
Chills	16 (20.25)	7 (29.17)	0.319
Cough	63 (79.75)	11 (45.83)	0.002
Sputum production	44 (55.70)	2 (8.33)	< 0.001
Chest tightness	9 (11.39)	2 (8.33)	0.997
Dyspnea	14 (17.72)	1 (4.17)	0.201
Sore throat	17 (21.52)	0	0.033
Fatigue	18 (22.78)	5 (20.83)	0.893
Headache	14 (17.72)	1 (4.17)	0.201
Myalgia	19 (24.05)	4 (16.67)	0.484
Abdominal pain	2 (2.53)	2 (8.33)	0.222
Diarrhea	2 (2.53)	2 (8.33)	0.222

Data are expressed as mean ± standard deviation or number of cases (percentage).

### Initial chest CT features

All 24 patients with COVID-19 had abnormal findings on initial chest CT. Of the 79 patients with influenza pneumonia, 64 had abnormal initial chest CT images and 15 had normal images. There were significant differences in central–peripheral distribution, superior–inferior distribution, anterior–posterior distribution, number of involved lobes, patches of GGO, GGO nodule, vascular enlargement in GGO, air bronchogram, bronchiolectasis within lesion, and interlobular septal thickening between the two groups, but no differences in other CT features (Table 2).

**Table 2:** Initial chest CT features in 79 patients with influenza pneumonia and 24 with COVID-19.

Findings	Influenza pneumonia (*n* = 79)	COVID-19 (*n* = 24)	*p*
**Laterality distribution (%)**			0.075
Left lobe	7 (8.86)	3 (12.50)	
Right lobe	8 (10.13)	3 (12.50)	
Bilateral lobe	49 (62.03)	18 (75.00)	
Negative CT	15 (18.99)	0	
**Central–peripheral distribution (%)**			0.000
Hilar	0	1 (4.17)	
Subpleural	23 (29.11)	19 (79.67)	
Random	41 (51.90)	4 (16.67)	
Negative CT	15 (18.99)	0	
**Superior–inferior distribution (%)**			0.013
Superior	5 (6.33)	0	
Inferior	27 (34.18)	17 (70.83)	
Radom	32 (40.51)	7 (29.17)	
Negative CT	15 (18.99)	0	
**Anterior–posterior distribution (%)**			0.002
Anterior	2 (3.13)	1 (4.17)	
Posterior	18 (28.13)	12 (50.00)	
Random	44 (68.76)	11 (45.83)	
Negative CT	15 (18.99)	0	
**Solitary lesion (%)**			0.631
Yes	2 (2.53)	5 (20.83)	
No	62 (78.48)	19 (79.17)	
Negative CT	15 (18.99)	0	
Number of involved lobes	2.68 (1.93)	3.54 (1.67)	0.048
Number of involved segments	6.57 (6.62)	8.04 (5.82)	0.195
Patches of GGO, n (%)	36 (45.57)	20 (83.33)	0.003
GGO nodule, n (%)	13 (16.46)	9 (37.5)	0.028
> 3 cm round like GGO, n (%)	0	1 (4.17)	0.233
Vascular enlargement in GGO, n (%)	7 (8.86)	16 (66.67)	0.000
Consolidation, n (%)	25 (31.65)	4 (16.67)	0.242
Air bronchogram, n (%)	20 (25.32)	15 (62.50)	0.001
Bronchiolectasis within lesion, n (%)	15 (18.99)	14 (58.33)	0.000
**Interlobular septal thickening, n (%)**			0.004
Outside GGO	8 (10.13)	2 (8.33)	
Within GGO	7 (8.86)	9 (37.50)	
Negative CT	64 (81.01)	13 (54.17)	
Crazy-paving pattern, n (%)	1 (1.27)	3 (12.50)	0.059
Solid nodule, n (%)	35 (44.30)	11 (45.83)	0.895
Halo sign nodule, n (%)	3 (3.80)	2 (8.33)	0.716
Reticular pattern, n (%)	6 (7.59)	3 (12.50)	0.739
Subpleural curvilinear line, n (%)	7 (8.86)	1 (4.17)	0.751
Linear opacity, n (%)	42 (53.16)	11 (45.83)	0.692
Cavity, n (%)	1 (1.27)	0	0.767
Pleural thickening, n (%)	15 (18.99)	2 (8.33)	0.359
Pleural effusion, n (%)	12 (15.19)	0	0.064
Lymphadenopathy, n (%)	10 (12.66)	2 (8.33)	0.830
Atelectasis, n (%)	2 (2.53)	0	0.587
Emphysema, n (%)	7 (8.86)	1 (4.17)	0.751
Pericardial effusion, n (%)	7 (8.86)	0	0.196

*GGO* ground-glass opacity.

**Figure 1:**
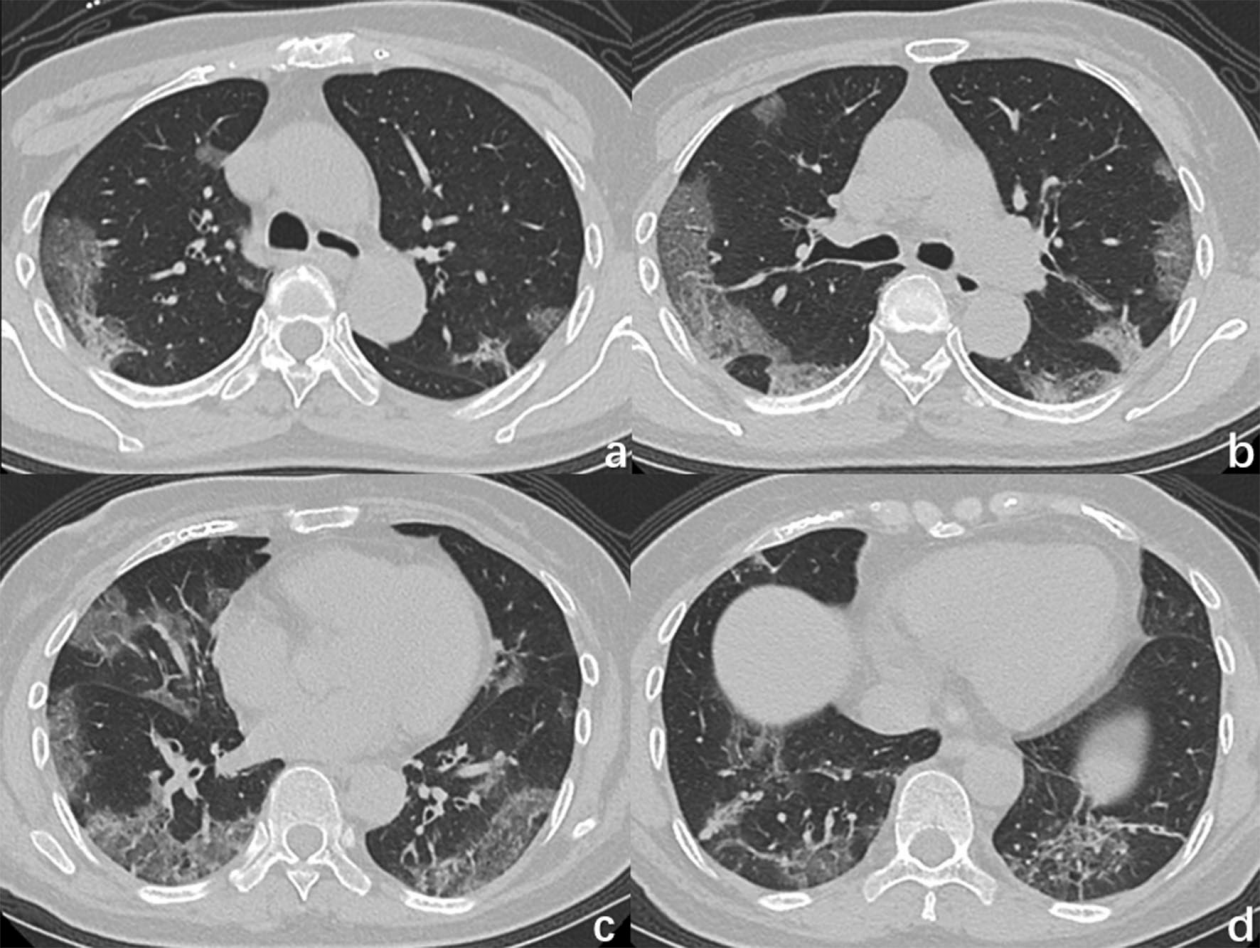
CT image of a 66-year-old man with COVID-19 infection, presenting fever with cough for 7 days, shows multiple GGOs in bilateral lung, mainly distributedin the subpleural areas, with reticular pattern (interlobular septal thickening).

**Figure 2:**
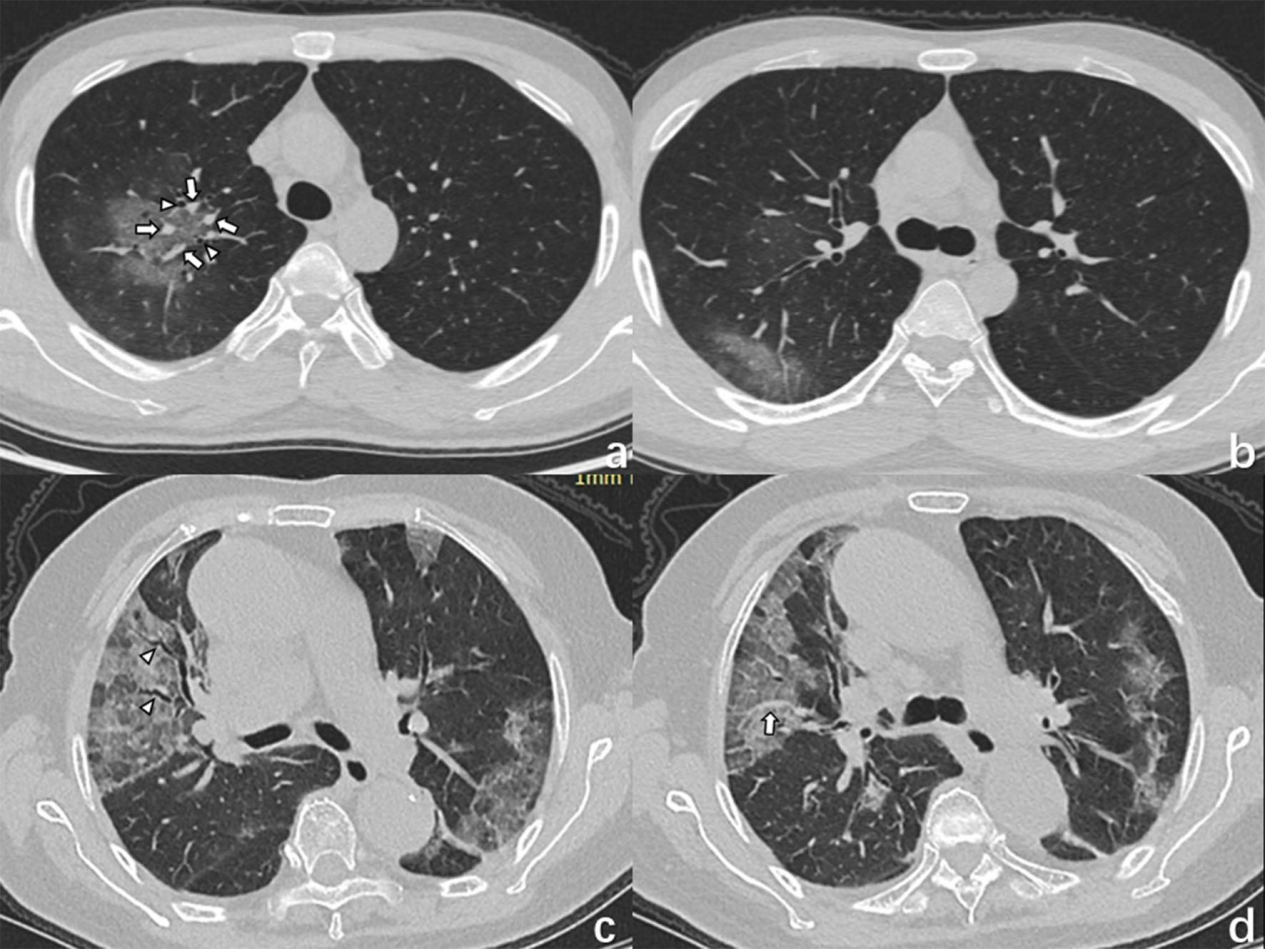
(**a**, **b**) A 33-year-old woman with COVID-19 infection, living in Wuhan, presented with fever, rhinocleisis, and dry cough. Axial (**a**) and coronal (**b**) CT images showing a subpleural mixed ground-glass opacity with multiple bronchiolectasis (triangles) and small vascular enlargement (arrow) in the right lower lobe. (**c**, **d**) A 47-year-old man with a COVID-19 infection, who traveled to Wuhan presented with fever. Axial (**c**) and coronal (**d**) CT images shows dominantly posterior- and inferior, multiple, GGOs with bronchiolectasis (triangles) and vascular enlargement (arrows).

**Figure 3:**
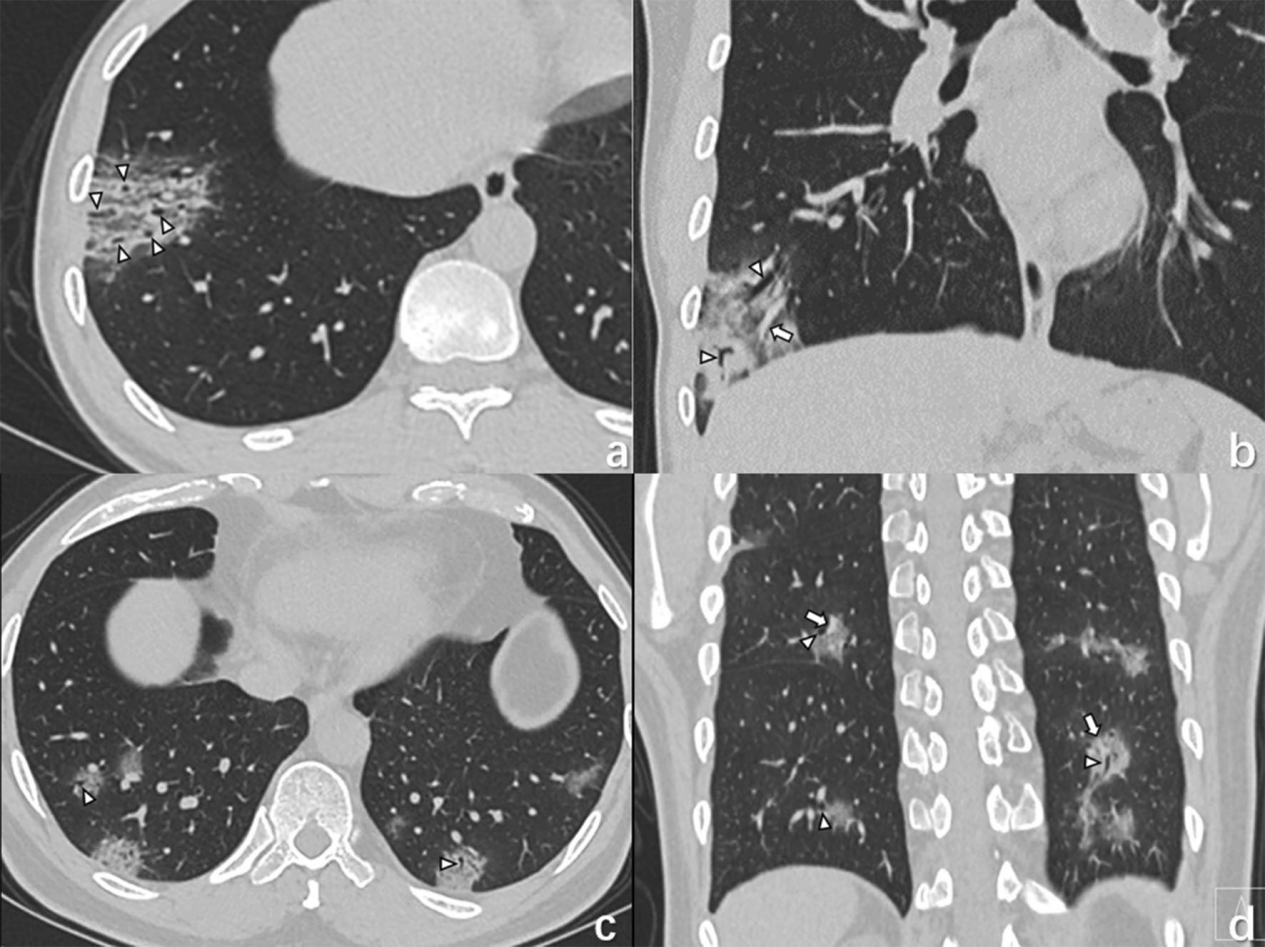
(**a**, **b**) A 35-year-old male with COVID-19 infection presenting fever and headache for 1 day. CT images shows small vascular enlargement (arrows) and bronchiolectasis (triangles) in GGO, as well as multiple GGOs in the subpleural areas of bilateral lung. (**c**, **d**) An 80-year old man with COVID-19 infection, who lives in Wuhan presented with fever and dry cough. CT images shows multiple patchy areas of GGOs as crazy-paving pattern with bronchiolectasis (triangles) and vascular enlargement (arrows).

Overall, lung lesions in patients with COVID-19 were most likely to appear in the subpleural, inferior, and posterior lung fields, whereas those in patients with influenza are mostly randomly distributed, without obvious regional distribution characteristics. Patients with COVID-19 were more likely to have isolated lesions than were patients with influenza. The following CT features were more prevalent in patients with COVID-19 than in patients with influenza (Figs. 1, 2, 3): patches of GGO, GGO nodule, vascular enlargement, air bronchogram, bronchiolectasis, and crazy-paving pattern. Interlobular septal thickening in patients with COVID-19 was more likely to appear within the GGO lesions, whereas in patients with influenza, it was more likely to appear outside the GGO lesions.

### Differentiating COVID-19 from influenza pneumonia

**Figure 4:**
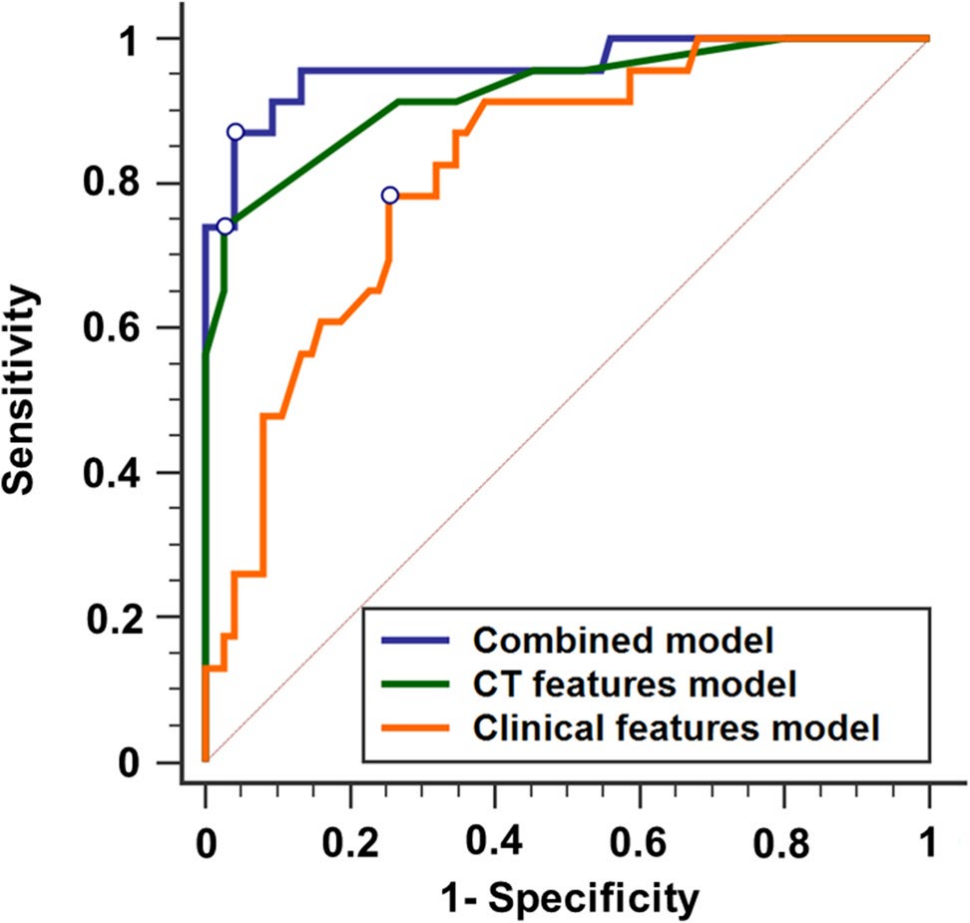
The performance characteristics of the logistic regression models for diagnosing patients with COVID-19 infection. Orange line, the model based on clinical variables; green line, the model based on CT features; purple line, the combined model with both clinical variables and CT features.

Firstly, univariate logistic analysis was used for screening the risk factors across clinical and CT features (Table 3). Univariate analysis demonstrated that the temperature (OR: 1.581, 95% CI 1.017–2.460), systolic pressure (OR: 1.033, 95% CI 1.003–1.064), cough (OR: 4.582, 95% CI 1.697–12.370) and sputum production (OR: 13.588, 95% CI 2.978–61.997) could distinguish COVID-19 from influenza pneumonia. The diagnostic sensitivity and specificity for the clinical features were 0.783 and 0.747, and the AUC value was 0.819 (Fig. 4).

**Table 3:** Univariate and multivariate analysis of clinical features and CT features for distinguishing COVID-19 and influenza pneumonia.

Variables	Univariate		Multivariate	
	*p*	OR (95% CI)	*p*	OR (95% CI)
**Clinical features**				
Temperature	0.042	1.581 (1.017–2.460)	0.723	1.111 (0.622–1.986)
Systolic pressure	0.031	1.033 (1.003–1.064)	0.033	1.043 (1.003–1.083)
Cough	0.003	4.582 (1.697–12.370)	0.337	1.773 (0.552–5.698)
Sputum production	0.001	13.588 (2.978–61.997)	0.005	11.525 (2.109–62.981)
**Anatomical distribution**				
Central–peripheral distribution	0.000	3.103 (1.798–5.355)	0.001	8.371 (2.287–30.645)
Superior–inferior distribution	0.023	2.121 (1.107–4.065)	0.727	1.292 (0.307–5.428)
Anterior–posterior distribution	0.005	2.804 (1.373–5.730)	0.369	2.065 (0.424–10.059)
**CT signs**				
GGO patchy	0.003	5.972 (1.870–19.075)	0.233	3.714 (0.431–32.028)
GGO nodule	0.032	3.046 (1.100–8.432)	0.029	9.243 (1.256–67.995)
Vascular enlargement in GGO	0.000	20.571 (6.514–64.966)	0.003	237.610 (6.784–8322.545)
Air bronchogram	0.001	4.917 (1.865–12.965)	0.094	16.214 (0.623–421.779)
Bronchiectasis within focus	0.000	5.973 (2.226–16.031)	0.037	100.120 (1.311–7529.702)
Interlobular septal thickening	0.002	2.403 (1.362–4.241)	0.959	1.035 (0.274–3.913)

Then, univariate analysis for the CT features was used for the further screening, and the variables including central–peripheral distribution (OR: 3.103, 95% CI 1.798–5.355), superior–inferior distribution (OR: 2.121, 95% CI 1.107–4.065), anterior–posterior distribution (OR: 2.804, 95% CI 1.373–5.730), patches of GGO (OR: 5.972, 95% CI 1.870–19.075), GGO nodule (OR: 3.046, 95% CI 1.100–8.432), vascular enlargement in GGO (OR: 20.571, 95% CI 6.514–64.966), air bronchogram (OR: 4.917, 95% CI 1.865–12.965), bronchiectasis within focus (OR: 5.973, 95% CI 2.226–16.031), interlobular septal thickening (OR: 2.403, 95% CI 1.362–4.241). The diagnostic sensitivity and specificity for the CT features were 0.750 and 0.962, and the AUC value was 0.927 (Fig. 4).

Finally, a multivariate logistic regression model combined the variables from the clinical features and CT features models. The combined model contained six features: systolic blood pressure (OR: 1.043, 95% CI 1.003–1.083), sputum production (OR: 11.525, 95% CI 2.109–62.981), vascular enlargement in the GGO (OR: 237.61, 95% CI 6.784–8322.545), GGO nodule (OR: 9.243, 95% CI 1.256–67.995), and central-peripheral distribution (OR: 8.371, 95% CI 2.287–30.645) and bronchiectasis within focus (OR: 100.120, 95% CI 1.311–7529.702). The diagnostic sensitivity and specificity for the combined features were 0.87 and 0.96, and the AUC value was 0.961 (Fig. 4).

## Discussion

Influenza virus and SARS-CoV-2 infection cause similar clinical symptoms, and both can cause viral pneumonia. RT-PCR is the most convenient and accurate method to identify the two respiratory viruses. However, there is a possibility of false negative in RT-PCR detection for various reasons, such as non-standard specimen collection. However, the chest CT findings of viral pneumonia are similar and overlapping, which is not enough to accurately distinguish the two kinds of viral pneumonia^12^.

Exposure history is very important, and there have been a lot of studies that have emphasized this issue^7,19–22^. In our study, all 24 patients with COVID-19 had exposure, whereas all 79 patients with influenza pneumonia had no exposure. However, since the time of the case reviewed was the beginning of the COVID-19 epidemic, Chengdu is more than 1000 km away from Wuhan, and the government implemented strict measures to restrict the movement of people, we believe that the exposure history of the cases in this study may have a serious bias. Based on this, this study did not consider the factor of exposure history when constructing the diagnostic model.

So far, many virological, pathogenesis, pathological and pathophysiological studies have gradually lifted the veil of COVID-19 for us^23–33^, and may also explain the differences in clinical and CT features between influenza pneumonia and COVID-19 showed by our study.

Angiotensin-converting enzyme 2 (ACE2) has been established as the functional host receptor for SARS-CoV-2^28–31^. ACE2 is abundantly expressed in a variety of cells residing in many different human organs. ACE2 is a pivotal counter-regulatory enzyme to ACE by the breakdown of angiotensin II, the central player in the renin–angiotensin–aldosterone system (RAAS) and the main substrate of ACE2^28–31^. In this study, systolic blood pressure was higher in patients with COVID-19 than in those with influenza pneumonia. We speculate that the reason is that ACE2 cannot bind to the substrate Ang II due to SARS-CoV-2 infection, and may accompany the virus to enter the cell interior, resulting in increased blood pressure and vasoconstriction.

Since mucus is a fundamental mechanism to defense against allergens and pathogens, its production increases in the respiratory tract in nearly every instance of airway inflammation. The cytokine storm in COVID-19 is particularly potent for the build-up of mucus due to the onset of several inflammatory cascades associated with mucus production^34^. Patients with COVID-19 had less sputum than patients with influenza pneumonia. This was caused by the pathological changes of COVID-19. In the lungs of COVID-19, terminal bronchi were obstructed by bronchial exudate, viscous secretions, and inflammatory exudate^23–27^. Such pathological changes rarely occur in influenza pneumonia.

The most obvious manifestation of COVID-19 in the lungs is the diffuse alveolar damage (DAD), with its congestion, proliferation and organising phases paradoxically juxtaposed, giving a picture of ‘temporal heterogeneity’^32,35,36^. The pathological changes in this stage include dilation and congestion of alveolar septal capillaries, exudate in alveolar cavity, and interstitial edema in interlobular septum. And vascular features were distinctive of COVID-19 and consisted of severe endothelial injury, widespread thrombosis with microangiopathy, alveolar capillary microthrombi, and neoangiogenesis^37^. This leads to patches of GGO, GGO nodule, vascular enlargement in GGO, air bronchogram, bronchiectasis within focus, and interlobular septal thickening on chest CT images of COVID-19.

The SARS-CoV-2 virus, with a diameter of 60–140 nm^19^, can easily reach the periphery of the lung and, as does SARS, bind to ACE2 receptors in the bronchioles, terminal bronchioles, and alveoli. This binding explains why the lesions associated with COVID-19 are mainly distributed in the periphery of the lung at least at the beginning of the infection. In contrast, the α2,6-linked sialic acid-bearing receptors, to which human influenza viruses preferentially bind, are abundant in the upper and lower respiratory tract of humans, particularly in tracheobronchial epithelium and type I alveolar cells^38^. Therefore, the influenza pneumonia lesions were not distributed mainly in the peripheral lung but in the central or the whole lung.

Several studies have compared the differences in CT features between influenza pneumonia and COVID-19^17,18^. But their results were not entirely satisfactory. Although there were not many cases in our study, we have observed many clinical variables and CT indicators and carried out regression analysis, and the results have high diagnostic performance, especially the integrated model combining CT features and clinical variables.

There are some limitations to our research. Our study was a single-center study. Not all patients were followed up to full recovery because some were later referred to infectious disease hospitals in our area. Pediatric, pregnant, and perinatal cases were not included because our hospital does not have a pediatric or an obstetrics and gynecology department. The small sample prevented validating the combined model. Therefore, we cannot be completely confident in the diagnostic performance of this model but hope to include more COVID-19 cases in the future. We plan to conduct a prospective, multicenter study, including more cases, to further determine the differences between COVID-19 and influenza pneumonia.

In conclusion, some CT features or clinical variables can differentiate COVID-19 from influenza pneumonia. Moreover, CT features combined with clinical variables had higher diagnostic performance.

## References

[1] WHO. Weekly epidemiological update—16 February 2021. https://www.who.int/publications/m/item/weekly-epidemiological-update---16-february-2021. (2021).

[2] Thompson WW, Shay DK, Weintraub E, Brammer L, Bridges CB, Cox NJ, Fukuda K (2004). Influenza-associated hospitalizations in the United States. JAMA.

[3] Lafond KE (2016). Global role and burden of influenza in pediatric respiratory hospitalizations, 1982–2012: A systematic analysis. PLoS Med.

[4] Uyeki TM (2017). Influenza. Ann. Intern. Med..

[5] Shang M, Blanton L, Brammer L, Olsen SJ, Fry AM (2018). Influenza-associated pediatric deaths in the United States, 2010–2016. Pediatrics.

[6] Paules C, Subbarao K (2017). Influenza. Lancet.

[7] Chen N (2020). Epidemiological and clinical characteristics of 99 cases of 2019 novel coronavirus pneumonia in Wuhan, China: A descriptive study. Lancet.

[8] Huang C (2020). Clinical features of patients infected with 2019 novel coronavirus in Wuhan, China. Lancet.

[9] Zou L (2020). SARS-CoV-2 viral load in upper respiratory specimens of infected patients. N. Engl. J. Med..

[10] Holshue ML (2020). First case of 2019 novel coronavirus in the United States. N. Engl. J. Med..

[11] Wang M (2020). A precision medicine approach to managing 2019 novel coronavirus pneumonia. Precis. Clin. Med..

[12] Stefanidis K (2021). Radiological, epidemiological and clinical patterns of pulmonary viral infections. Eur. J. Radiol..

[13] Koo HJ, Lim S, Choe J, Choi SH, Sung H, Do KH (2018). Radiographic and CT features of viral pneumonia. Radiographics.

[14] Miller WT, Mickus TJ, Barbosa E, Mullin C, Van Deerlin VM, Shiley KT (2011). CT of viral lower respiratory tract infections in adults: Comparison among viral organisms and between viral and bacterial infections. AJR Am. J. Roentgenol..

[15] Wan S, Li M, Ye Z, Yang C, Cai Q, Duan S, Song B (2020). CT manifestations and clinical characteristics of 1115 patients with coronavirus disease 2019 (COVID-19): A systematic review and meta-analysis. Acad. Radiol..

[16] Ye Z, Zhang Y, Wang Y, Huang Z, Song B (2020). Chest CT manifestations of new coronavirus disease 2019 (COVID-19): A pictorial review. Eur. Radiol..

[17] Wang H, Wei R, Rao G, Zhu J, Song B (2020). Characteristic CT findings distinguishing 2019 novel coronavirus disease (COVID-19) from influenza pneumonia. Eur. Radiol..

[18] Lin L, Fu G, Chen S, Tao J, Qian A, Yang Y, Wang M (2021). CT manifestations of coronavirus disease (COVID-19) pneumonia and influenza virus pneumonia: A comparative study. AJR Am. J. Roentgenol..

[19] Zhu N (2020). A novel coronavirus from patients with pneumonia in China, 2019. N. Engl. J. Med..

[20] Rothe C (2020). Transmission of 2019-nCoV infection from an asymptomatic contact in Germany. N. Engl. J. Med..

[21] Li Q (2020). Early transmission dynamics in Wuhan, China, of novel coronavirus-infected pneumonia. N. Engl. J. Med..

[22] Tian, S. *et al.* Characteristics of COVID-19 infection in Beijing. *J. Infect*. **80**, 401–406 (2020). 10.1016/j.jinf.2020.02.018 PMC7102527 32112886

[23] Pandey P, Agarwal RS (2020). Lung pathology in COVID-19: A systematic review. Int. J. Appl. Basic Med. Res..

[24] Chen W, Pan JY (2021). Anatomical and pathological observation and analysis of SARS and COVID-19: Microthrombosis is the main cause of death. Biol. Proced. Online.

[25] Zarrilli, G. *et al.* The immunopathological and histological landscape of COVID-19-mediated lung injury. *Int. J. Mol. Sci*. **22,** 974 (2021). 10.3390/ijms22020974 PMC7835817 33478107

[26] Montero-Fernández, M. A. & Pardo-Garcia, R. Histopathology features of the lung in COVID-19 patients. *Diagn. Histopathol. (Oxf.)* .**27**, 123-127(2021). 10.1016/j.mpdhp.2020.11.009 PMC7717771 33312229

[27] Balaky S (2020). A comprehensive review of histopathological findings of infections induced by COVID-19. Cell Mol. Biol. (Noisy-le-grand).

[28] Bourgonje AR (2020). Angiotensin-converting enzyme 2 (ACE2), SARS-CoV-2 and the pathophysiology of coronavirus disease 2019 (COVID-19). J. Pathol..

[29] Datta PK, Liu F, Fischer T, Rappaport J, Qin X (2020). SARS-CoV-2 pandemic and research gaps: Understanding SARS-CoV-2 interaction with the ACE2 receptor and implications for therapy. Theranostics.

[30] Groß S, Jahn C, Cushman S, Bär C, Thum T (2020). SARS-CoV-2 receptor ACE2-dependent implications on the cardiovascular system: From basic science to clinical implications. J. Mol. Cell Cardiol..

[31] South AM, Diz DI, Chappell MC (2020). COVID-19, ACE2, and the cardiovascular consequences. Am. J. Physiol. Heart Circ. Physiol..

[32] Bösmüller, H., Matter, M., Fend, F. & Tzankov, A. The pulmonary pathology of COVID-19. *Virchows Arch*. **19**, 1-14 (2021). 10.1007/s00428-021-03053-1 PMC7892326 33604758

[33] Lopes-Pacheco M (2021). Pathogenesis of multiple organ injury in COVID-19 and potential therapeutic strategies. Front. Physiol..

[34] Khan MA (2021). Cytokine storm and mucus hypersecretion in COVID-19: Review of mechanisms. J. Inflamm. Res..

[35] Borczuk AC (2020). COVID-19 pulmonary pathology: A multi-institutional autopsy cohort from Italy and New York City. Mod. Pathol..

[36] Schaefer IM, Padera RF, Solomon IH, Kanjilal S, Hammer MM, Hornick JL, Sholl LM (2020). In situ detection of SARS-CoV-2 in lungs and airways of patients with COVID-19. Mod. Pathol..

[37] Ackermann M (2020). Pulmonary vascular endothelialitis, thrombosis, and angiogenesis in COVID-19. N. Engl. J. Med..

[38] Rogers GN, Paulson JC (1983). Receptor determinants of human and animal influenza virus isolates: Differences in receptor specificity of the H3 hemagglutinin based on species of origin. Virology.

